# Association of atherogenic index of plasma with early-onset post-stroke depression: a prospective study

**DOI:** 10.3389/fpsyt.2025.1563289

**Published:** 2025-07-25

**Authors:** Mingzhu Deng, Kangping Song, Guohua He, Wei Zhao, Wei Xu, Tieqiao Feng, Sufen Chen, Yangping Tong, Yanqing Fei, Zhen Wang, Fangyi Li

**Affiliations:** ^1^ Department of Neurology, The Second People’s Hospital of Hunan Province (Brain Hospital of Hunan Province), Changsha, Hunan, China; ^2^ Department of Neurology, The Affiliated Changsha Central Hospital, Hengyang Medical School, University of South China, Changsha, Hunan, China

**Keywords:** post-stroke depression, acute ischemic stroke, atherogenic index of plasma, abnormal lipid metabolism, depression

## Abstract

**Background:**

The atherogenic index of plasma (AIP) is a newly developed marker of lipids that has strong prognostic value in people with cardiovascular disease. Nevertheless, few studies concern the relationship between AIP and early-onset post-stroke depression (PSD).

**Methods:**

After two weeks of acute ischemic stroke (AIS), early-onset PSD was identified. The Hamilton Depression Scale-17 items (HAMD-17) was used to assess the severity of depression. Patients with HAMD-17 scores ≥7 were divided into an early-onset PSD group. Spearman rank correlation analysis was employed to evaluate the associations between AIP and HAMD scores across all patients. Logistic regression analysis was conducted to investigate the associations between the AIP and early-onset PSD. Receiver operating characteristic curve (ROC) analysis was used to determine the predictive value of the AIP for early-onset PSD.

**Results:**

Among the 667 recruited patients, a total of 225 (33.73%) patients were diagnosed with early-onset PSD. The AIP showed a positive correlation with the HAMD-17 scores (r=0.567, P<0.001). A binary logistic regression model demonstrated that the AIP (odds ratio [OR], 1.843; 95% confidence interval [CI] 1.650–2.558, P<0.001) was an independent factor for early-onset PSD. The AIP for early-onset PSD had an area under the curve (AUC) value of 0.785.

**Conclusions:**

Our study indicates that the AIP may serve as an independent risk factor for early-onset PSD, offering insights for the prevention and management of prognosis in affected patients.

## Introduction

Post-stroke depression (PSD) is a severe and frequent complication of mental disorders following stroke (1), which is characterized by low mood, loss of interest, and even suicidal tendencies (2). The prevalence of PSD ranges from 18% to 33% (3, 4). Early-onset PSD refers to patients exhibiting depression within two weeks after acute stroke onset (5, 6). Early-onset PSD is characterized by a higher incidence of depressive symptoms and is strongly related to an increased risk of negative consequences compared to late-onset PSD (7). Furthermore, PSD correlates with elevated mortality rates and imposes further burdens on the families of affected individuals and society in general (8). Therefore, the early identification and treatment of early-onset PSD are of great enormous significance.

Atherosclerosis is the pathogenesis of many cardiovascular and cerebrovascular diseases (9). Dyslipidemia is the most important risk factor for atherosclerosis and a major pathogenic factor for cerebrovascular diseases (10). Furthermore, lipid metabolism is significantly associated with stroke outcomes. A systematic review and meta-analysis of prospective cohort studies showed that lower lipid levels may decrease the risk of worsening ischemic stroke while preserving potential benefits in reducing hemorrhagic stroke risk (11). A recent study indicates that lipid metabolism may be associated with embolization recurrence after alteplase treatment (12). Several lipid markers, including non-high-density lipoprotein cholesterol, triglyceride (TG), high-density lipoprotein cholesterol (HDL-C), total cholesterol (TC), and low-density lipoprotein cholesterol (LDL-C), have been used to assess the risk of stroke outcomes (13–16). Nonetheless, the conventional singular index of blood lipids as a measure of atherosclerosis and cerebrovascular disorders is still contentious, and the prognostic significance of these indicators remains limited. The atherogenic index of plasma (AIP) is determined by the logarithmic ratio of TG to HDL-C levels (17). A previous study demonstrated that AIP effectively elucidates the interactions among various lipid components and serves as a sensitive indicator for evaluating lipid disorders (16). Multiple epidemiological studies have indicated a significant association between AIP and coronary heart disease (18). Elevated AIP was associated with increased risk of ischemic stroke and poor outcomes in stroke patients (19, 20). Moreover, research has indicated a correlation between AIP and the occurrence of depression, suggesting that elevated levels of AIP may heighten the risk of developing depression (21). A recent study has indicated that AIP could serve as a promising predictor for the risk of developing PSD (22). Numerous investigations have demonstrated a complex link between depression and atherosclerosis, two ostensibly distinct illnesses that have common underlying pathophysiological mechanisms (23, 24). Epidemiological studies consistently indicate an increased risk of myocardial infarction and stroke in individuals with depression (25). This correlation is partly explained by the shared mechanisms of inflammation, oxidative stress, and endothelial dysfunction (26). So far, the relationship between AIP and early-onset PSD is still unclear. Based on the above research, we speculate that AIP may be closely related to the occurrence and development of early-onset PSD.

Early-onset PSD is characterized by a greater prevalence of depressive symptoms and is substantially associated with a greater chance of poor outcomes (27). Thus, it is of significant value to detect predictive factors for early-onset PSD. In this study, we aimed to investigate the association between AIP and early-onset PSD and to explore whether AIP can serve as a predictive indicator for early-onset PSD.

## Materials and methods

### Study design and participants

**Figure 1:**
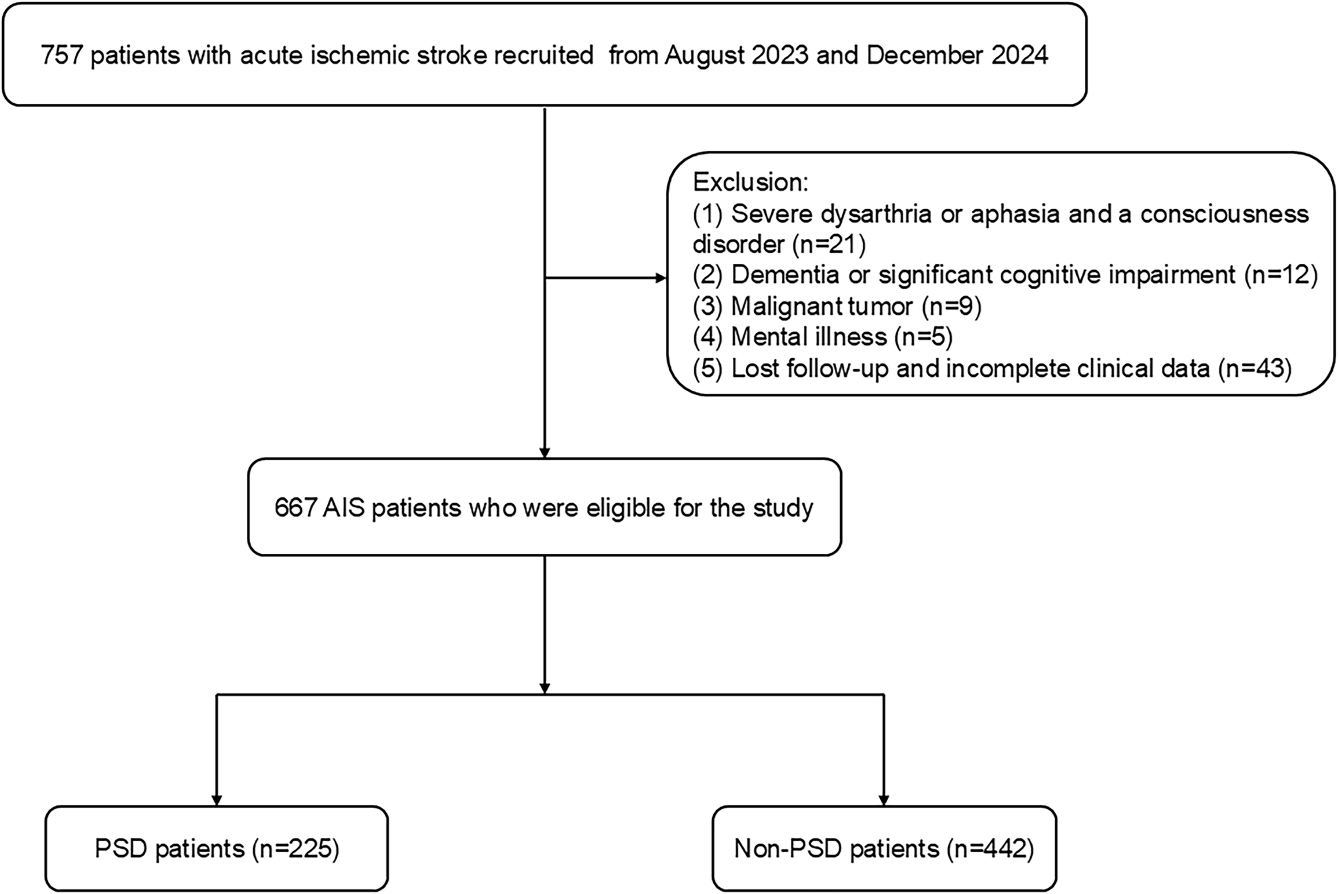
The flow diagram for the investigation. AIS, acute ischemic stroke; PSD, post-stroke depression.

Patients with acute ischemic stroke (AIS) were prospectively enrolled at Changsha Central Hospital from August 2023 to December 2024. The Ethics Committee at Changsha Central Hospital authorized this study. AIS was diagnosed using head imaging techniques, including CT and MRI, based on the 2018 Chinese guidelines for the diagnosis and treatment of acute ischemic stroke (28). The diagnostic criteria were as follows: 1) acute onset; 2) focal neurological deficits (weakness or numbness of one side of the face or limb, language disorder, etc.), a few of which are global neurological deficits; 3) imaging liability lesions or symptoms/signs for more than 24 h; 4) exclusion of non-vascular causes; and 5) brain CT/MRI exclusion of cerebral hemorrhage. The following patients met the inclusion criteria: (1) those who met the diagnostic criteria for ischemic stroke as stated in the Chinese guidelines for diagnosis and treatment of acute ischemic stroke 2018. (2) those who were between the ages of 18 and 85; and (3) those who were admitted to the hospital within 72 hours of the stroke onset. Patients who had any of the following criteria were excluded: (1) those who had severe dysarthria or aphasia and a consciousness disorder that made it hard for them to complete tests and questionnaires; (2) those who had dementia or significant cognitive impairment before the stroke; (3) those who had severe heart, liver, or renal insufficiency; (4) those who had a mental illness, such as depression, or were using psychotropic drugs before the stroke; (5) those who had a history of other diseases in the central nervous system, like Parkinson's disease or epilepsy; (6) those who had malignant tumors; and (7) those who lost follow-up (including require psychiatric medications such as antidepressants during hospitalization and after discharge) and incomplete clinical data. We recruited 667 AIS patients between August 2023 and December 2024 (**Figure 1**).

### Data collection

On admission, all subjects had conventional assessments of their age, sex, BMI, and vascular risk factors (diabetes mellitus, hypertension, atrial fibrillation, coronary artery disease, current smoking and drinking, and laboratory tests). Patients were considered current smokers if they had smoked at least 10 cigarettes a day over the previous five years. Patients were considered current drinkers if they had drunk consistently for five years and consumed at least 20 grams of ethanol per day. At the time of admission, experienced neurologists assessed the severity of the stroke using the National Institutes of Health Stroke Scale (NIHSS). Within 24 hours following admission, NIHSS scores were evaluated. The Barthel Index (BI) scores were assessed at discharge. Furthermore, at the one-month follow-up, the functional outcome was evaluated based on the modified Rankin Scale (mRS) score. Computed tomography, magnetic resonance imaging, echocardiography, electrocardiogram, carotid ultrasonography, and transcranial Doppler were used to identify the lesion site and stroke subtype.

Expert neurologists and psychiatrists conducted the clinical evaluations in a blinded manner. After two weeks of AIS, individuals with early-onset PSD were diagnosed by qualified neurologists and psychiatrists in accordance with the Diagnostic and Statistical Manual of Mental Disorders, 5th Edition (DSM-V) (29). The Hamilton Depression Scale 17 items (HAMD-17) were used to assess the severity of depressed symptoms, and they were administered by trained professionals. Patients with HAMD-17 scores <7 were enrolled in the non-PSD group. Patients with HAMD-17 scores ≥ 7 were enrolled in the early-onset PSD group. Mild depression is defined by a score of 7–17, moderate depression by 18–23, and severe depression by greater than 24 (30–32). Patients were divided into categories based on their scores: mild, moderate, or severe early-onset PSD.

All patients whose stroke occurred within 72 hours had their blood drawn at 6 or 7 a.m. the day after they had fasted for at least 8 hours. The automated hematology analyzer (BZ6800, CHINA) was utilized for standard blood tests, which involved the counts of white blood cells (WBC). An automated analyzer (HITACHI 7600, JAPAN) was used to perform a standard biochemical examination. The tests included measurements of fasting blood glucose (FBG), TG, TC, HDL-C, and LDL-C. Each blood sample was tested three times. All the indicators were tested using commercial kits, which were operated by qualified professionals in accordance with the specifications. The following formula was used to calculate AIP: Log [TG (mmol/L) / HDL-C (mmol/L)] (33).

### Statistical analysis

The target sample size was prospectively calculated using a two-sample independent t-test model in G*Power 3.1, based on the following clinically and methodologically informed parameters: Effect Size (Cohen's d): 0.5; α (Type I Error): 0.05 (two-tailed); Target Power (1-β): 80%. Data analysis was conducted using SPSS 25.0 (IBM SPSS Statistics software, Version 25.0). The Kolmogorov-Smirnov test was employed to assess if the distribution of all the data was normal. If the continuous variables were regularly distributed, they were shown as mean ± SD; if not, they were shown as median (quartile). For categorical factors, results are shown as percentages. The chi-squared test or Fisher's exact test was employed to analyze categorical data, while the Student's t-test or Mann-Whitney U test was utilized to assess disparities in baseline features of continuous variables among groups, followed by Bonferroni *post-hoc* tests, respectively. We used the box plots to show the distribution of variables (including AIP, age, FBG, TG, TC, and HDL-C) among early-onset PSD of different severity. Analyses using the Spearman rank correlation were carried out to investigate the correlation that exists between the variables (including AIP, age, FBG, TG, TC, and HDL-C) and HAMD scores in all patients. Stepwise multiple logistic regression analysis was performed to detect the independent influence of selected variables on patients with early-onset PSD. Collinearity diagnostics were used to detect the presence of multicollinearity between independent variables. However, TG and HDL-C were not included in the model because of collinearity with the AIP. The selected variables included age, male, NIHSS score, mRS score, BI score, FBG, TC, and AIP. Furthermore, subgroup analyses and interaction test. To evaluate the overall discriminative power of AIP for early-onset PSD, a receiver operating characteristic (ROC) curve was created using a MedCalc 15.6.0 (MedCalc Software Acacialaan 22, B-8400 Ostend, Belgium) packet program. A two-tailed value of P<0.05 was considered significant.

## Results

### Non-PSD and early-onset PSD clinical and demographic characteristics

**Figure 2:**
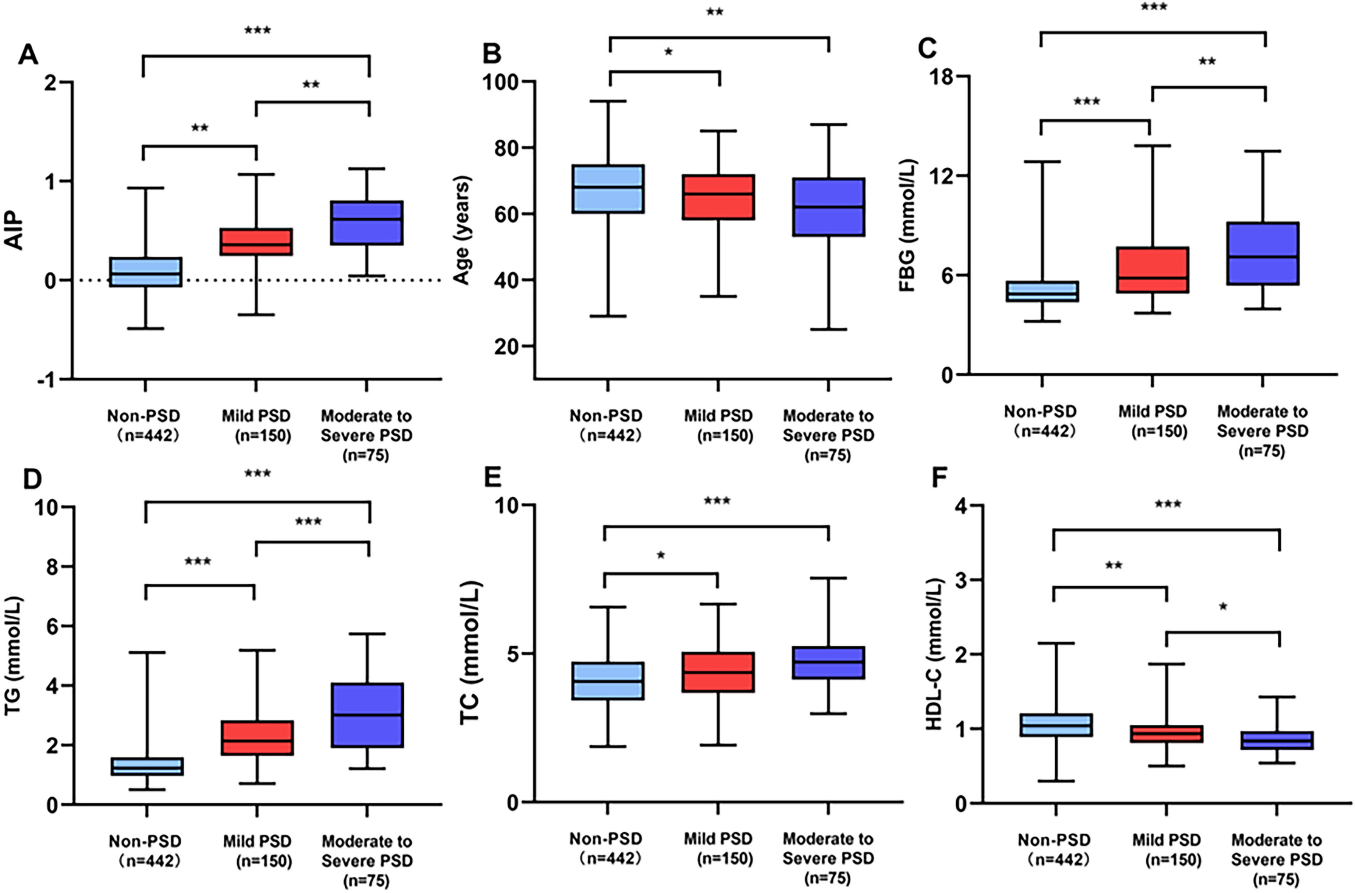
Comparison of AIP **(A)**, age **(B)**, FBG **(C)**, TG **(D)**, TC **(E)**, and HDL-C **(F)** among early-onset PSD of different severity. ***P<0.001, **P<0.01, *P<0.05.

**Table 1** provides a detailed exhibition of the clinical and demographic features. In this study, the early-onset PSD group was observed in 225 patients (33.73%), and the non-PSD group was observed in 442 patients (66.27%). The early-onset PSD group showed significantly lower percentages of age (P<0.001), male patients (P<0.001), BI score (P<0.001), and HDL-C (P<0.001) than the non-PSD group, but significantly higher NIHSS score (P=0.001), mRS score (P=0.002), HAMD-17 score (P<0.001), FBG (P<0.001), TG (P<0.001), TC (P<0.001), and AIP (P<0.001) than the non-PSD group. Furthermore, **Figure 2** showed that AIP, age, FBG, TG, TC, and HDL-C were compared among early-onset PSD with different levels of severity.

**Table 1:** Characteristics of patients in the non-PSD and PSD groups.

Variable	Total (n=667)	PSD (n=225)	Non-PSD (n=442)	P
Demographic characteristics				
Age, years	66.14 ± 11.41	63.47 ± 11.74	67.5 ± 10.99	<0.001
Male, n (%)	443 (66.42)	129 (57.33)	314 (71.04)	<0.001
BMI, kg/m^2^	23.58 ± 3.36	23.38 ± 3.41	23.69 ± 3.34	0.095
Vascular risk factors, n (%)				
Hypertension	536 (80.36)	187 (83.11)	349 (78.96)	0.202
Diabetes mellitus	169 (25.34)	58 (25.78)	111 (25.11)	0.852
Coronary artery disease	122 (18.29)	33 (14.67)	89 (20.14)	0.084
Current smoking	288 (43.18)	94 (41.78)	194 (43.89)	0.602
Current drinking	178 (26.69)	57 (25.33)	121 (27.38)	0.573
Stroke subtype, n (%)				0.989
LAA	176 (26.39)	60 (26.67)	116 (26.24)	
SAO	308 (46.18)	105 (46.67)	203 (45.93)	
CE	121 (18.14)	41 (18.22)	80 (18.10)	
SOE	27 (4.05)	8 (3.56)	19 (4.30)	
SUE	35 (5.25)	11 (4.89)	24 (5.43)	
Lesion location, n (%)				
Frontal lobe	50 (7.50)	19 (8.44)	31 (6.97)	0.492
Temporal lobe	24 (3.60)	9 (4.00)	15 (3.37)	0.679
Parietal lobe	55 (8.25)	19 (8.44)	36 (8.09)	0.875
Occipital lobe	21 (3.15)	6 (2.67)	15 (3.37)	0.621
Basal ganglia	276 (41.38)	93 (41.33)	183 (41.12)	0.958
Thalamus	44 (6.60)	14 (6.22)	30 (6.74)	0.853
Brainstem	140 (20.99)	47 (20.89)	93 (20.90)	0.998
Cerebellum	60 (9.00)	18 (8.00)	42 (9.44)	0.538
Medication use history, n (%)				
Previous antiplatelet drugs	100	30 (13.33)	70 (15.83)	0.392
Previous lipid-altering drugs	82	29 (12.89)	53 (11.99)	0.856
Previous antihypertension drugs	494	172 (76.44)	322 (72.85)	0.938
Previous hypoglycemic drugs	162	56 (24.89)	106 (23.98)	0.760
Neuropsychological evaluation				
NIHSS score, median (IQR)	2 (1-5)	3 (1-6)	2 (1-4)	0.001
mRS score, median (IQR)	1 (1-2)	2 (1-4)	1 (1-2)	0.002
BI score, median (IQR)	85 (75-100)	80 (70-100)	94 (73-100)	<0.001
HAMD-17score, median (IQR)	5 (3-12)	16 (12-19)	4 (2-5)	<0.001
laboratory data				
WBC (×10^9^/L)	6.88 ± 1.97	6.89 ± 1.98	6.87 ± 1.97	0.912
FBG (mmol/L)	5.21 (4.52-6.70)	6.17 (5.03-8.53)	4.86 (4.35-5.75)	<0.001
TG (mmol/L)	1.51 (1.10-2.20)	2.42 (1.74-3.41)	1.24 (0.97-1.62)	<0.001
TC (mmol/L)	4.31 (3.55-4.94)	4.57 (3.85-5.27)	4.16 (3.46-4.87)	<0.001
HDL-C (mmol/L)	1.02 ± 0.26	0.94 ± 0.27	1.07 ± 0.25	<0.001
LDL-C (mmol/L)	2.56 ± 0.83	2.72 ± 0.89	2.71 ± 0.78	0.165
AIP	0.17 (001-0.38)	0.42 (0.26-0.60)	0.06 (-0.07-0.24)	<0.001

NIHSS, National Institutes of Health Stroke Scale; mRS, modified Rankin Scale; BI, Barthel Index; HAMD-17, Hamilton depression scale 17 items; LAA, large-artery atherosclerosis; SAO, small-artery occlusion; CE, cardioembolism; SOE, stroke of other determined etiology; SUE, stroke of undetermined etiology; WBC, white blood cell; FBG, fasting blood glucose; TG, triglyceride; TC, total cholesterol; HDL-C, high-density lipoprotein cholesterol; LDL-C, low-density lipoprotein cholesterol; AIP, atherogenic index of plasma.

### Correlation analysis among the factors and the HAMD scores in all patients

**Figure 3:**
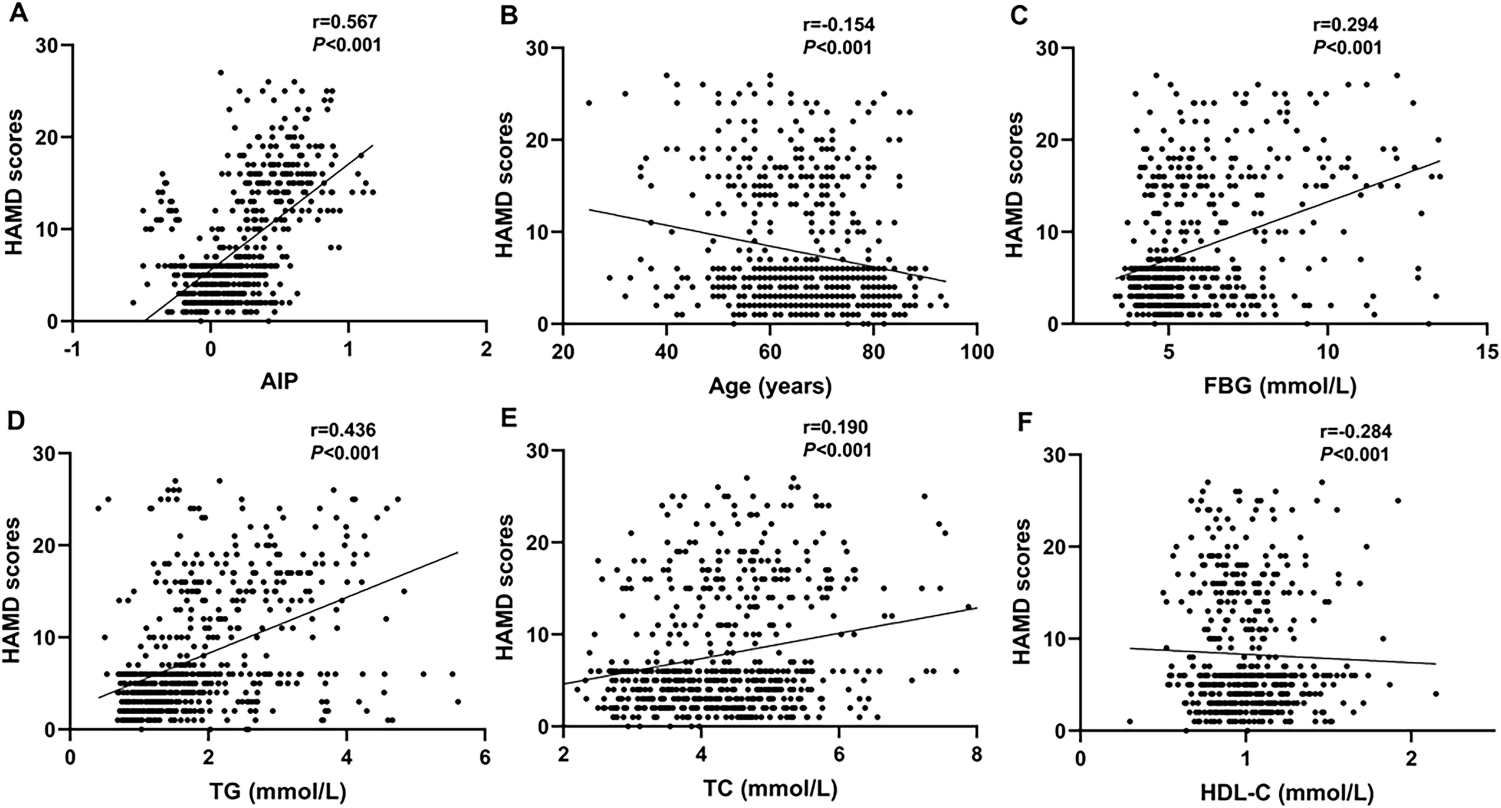
Scatter plots showing that the AIP (r=0.567, P<0.001; **(A)**, FBG (r=0.294, P<0.001; **(C)**, TG (r=0.436, P<0.001; **(D)**, and TC (r=0.190, P<0.001; **(E)** were positively correlated with the HAMD scores. The age (r=-0.154, P<0.001; **(B)** and HDL-C (r=-0.284, P<0.001; **(F)** were negatively correlation with the HAMD scores.

The HAMD-17 scores showed a positive correlation with the AIP (r=0.567, P<0.001), FBG (r=0.294, P<0.001), TG (r=0.436, P<0.001), and TC (r=0.190, P<0.001). Nevertheless, the HAMD scores showed a negative correlation with age (r=-0.154, P<0.001) and HDL-C (r=-0.284, P<0.001) (**Figure 3**).

### Analysis of logistic regression for risk factors associated with early-onset PSD

To investigate the risk factors connected with early-onset PSD, logistic regression models were employed. To identify independent risk factors for early-onset PSD, we used the binary logistic regression model to analyze variables of statistical significance mentioned in baseline data of the two groups (**Table 1**). In our study, there were no statistically significant differences in comorbid conditions or medication use history between the two groups at baseline. The outcomes of crude models for early-onset PSD are shown in **Table 2**. However, TG and HDL-C were not included in the model because of collinearity with the AIP. AIP (OR, 1.843; 95% CI 1.650-2.558, P<0.001) was revealed to be an independent predictor for early-onset PSD after adjusting for all other confounders. Furthermore, the AIP was utilized as a classification variable based on tertiles. Following the adjustment for confounding variables, patients exhibiting elevated AIP levels (T3 vs T1; OR, 1.915; 95% CI, 1.425−2.337, P=0.003) demonstrated a heightened risk of early-onset PSD (**Table 3**).

**Table 2:** Logistic regression analysis for risk factors with early-onset PSD.

Variable	OR (95% CI)	P	Adjusted OR (95% CI)	P
Age	0.969 (0.955-0.983)	0.003	0.705 (0.515-0.915)	0.726
Male	0.715 (0.515-0.913)	0.009	0.695 (0.508-0.836)	0.059
NIHSS score	1.943 (1.895-1.994)	0.028	1.686 (1.491-1.868)	0.897
mRS score	1.516 (1.321-1.915)	0.005	1.491 (0.947-2.034)	0.081
BI score	1.385 (1.195-1.537)	0.032	1.186 (1.005-1.342)	0.236
FBG	1.477 (1.337-1.631)	0.002	1.318 (1.115-1.584)	0.321
TG	1.943 (1.072-2.509)	<0.001		
TC	1.484 (1.270-1.739)	0.015	1.355 (1.158-1.533)	0.115
HDL-C	0.824 (0.659-0.959)	<0.001		
AIP	2.346 (1.715-2.892)	<0.001	1.843 (1.650-2.558)	<0.001

**Table 3:** Association between AIP and early-onset PSD.

Variable	OR (95% CI)	P	Adjusted OR (95% CI)^a^	P
AIP ternary classification				
T1	Reference		Reference	
T2	1.759 (1.417-2.006)	<0.001	1.515 (1.217-1.935)	0.004
T3	2.314 (1.876-2.918)	<0.001	1.915 (1.425-2.373)	0.003

a Model: adjusted for age, male, initial NIHSS score, mRS score, BI score, FBG, TG, TC, and HDL-C.

### Subgroup analyses and interaction test

To eliminate the influence of hypertension, diabetes, and other comorbidities, we also conducted stratified analyses to investigate whether the association between the AIP and early-onset PSD remained consistent across different subgroups (**Supplementary Figure 1**). Our subgroup analyses revealed that the positive correlation was not significantly altered by stratification variables including hypertension, diabetes mellitus, atrial fibrillation, coronary artery disease, smoking status, and alcohol use. There was no statistically significant difference in the relationship between the AIP and early-onset PSD across these strata, as indicated by the interaction test P*>*0.05, suggesting that covariates have no significant effect on this association.

### The overall discriminative ability for early-onset PSD was evaluated using ROC curves for the AIP

**Figure 4:**
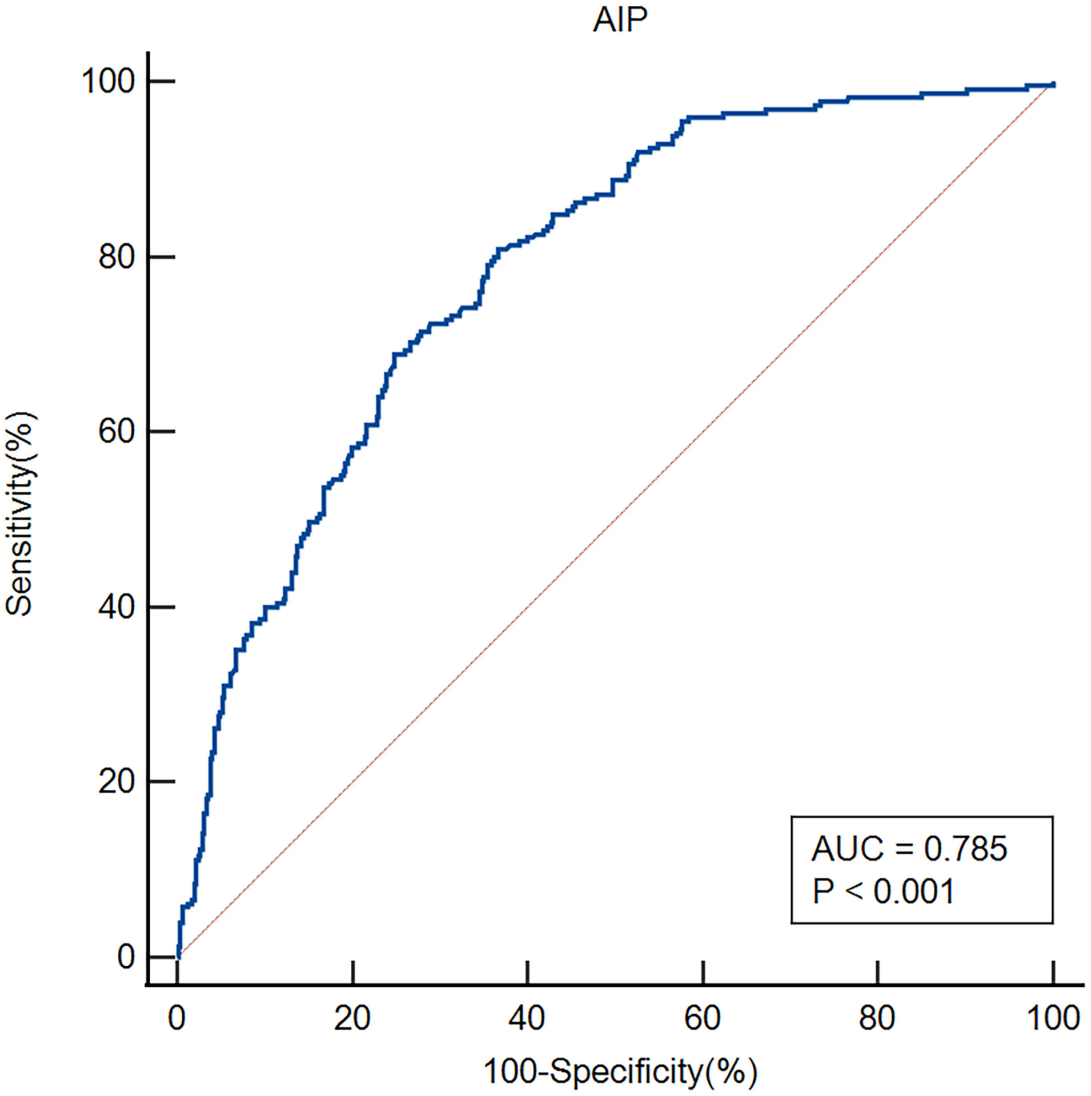
ROC analysis revealed that the AIP exhibited respectable early-onset PSD discriminating power, with AUC values of 0.785.

ROC curves were employed to evaluate the AIP's overall capacity to distinguish early-onset PSD (**Figure 4**). The AIP's area under the curve (AUC) for determining early-onset PSD was 0.785 (95% CI, 0.752–0.816; P<0.001); the cut-off value was 0.18, with a sensitivity of 80.89% and a specificity of 63.35%.

## Discussion

Researchers have currently identified various risk factors, such as gender, age, inflammatory cytokines, lesion localization, stroke severity, a history of depression, and symptomatic plaque enhancement, as being associated with PSD (4, 34). Additionally, recent research has demonstrated that early-onset PSD is closely associated with homocysteine, fibrinogen, and the ratio of non-high-density lipoprotein cholesterol to high-density lipoprotein cholesterol (1, 33). However, the relationship between AIP and early-onset PSD remains unknown. This research represents the investigation into the relationship between AIP and early-onset PSD. This investigation yielded some novel discoveries. Initially, patients in the early-onset PSD group had higher AIP, NIHSS, and mRS scores and a lower BI score than patients in the non-PSD group. The HAMD-17 scores showed a positive correlation with the AIP. Additionally, the logistic regression model indicated that AIP was an independent risk factor for early-onset PSD. Previous studies have shown that gender, age, lesion location, and stroke severity are associated with post-stroke depression. Lastly, according to ROC analysis, the AIP exhibited respectable early-onset PSD discriminating power. The findings indicate that a higher AIP correlates with early-onset PSD.

In our study, 225 patients (33.73%) experienced early-onset PSD, and the proportion was consistent with the results of previous studies (1, 6, 32). Prior research indicates that stroke survivors with early-onset depression (within 3 months after stroke) are at high risk for remaining depressed and make up two-thirds of the incident cases during 1 year after stroke. This study highlights the need for ongoing clinical monitoring of patients depressed shortly after stroke (35). In comparison to the non-PSD group, we found that the early-onset PSD group had a lower BI score and a higher NIHSS and mRS score, indicating a higher severity of stroke and disability. Depressive symptoms and other psychological issues might be exacerbated by unfavorable physical circumstances (36). Female stroke patients were found to be more likely to experience PSD than their male counterparts (22, 37, 38), which is consistent with our findings. Social variables like exposure to gender-specific stressors, psychological factors like gender-specific symptom profiles, and physiological aspects like sex hormones and genetic variations are some possible answers (39). There was also a higher likelihood of "reactive" depressed symptoms and more severe depressive symptoms in female patients following the stroke occurrence (40). The AIP had a positive relationship with the severity of early-onset PSD, which is consistent with previous studies (22, 37, 38). After adjusting for confounding variables, a binary logistic regression model showed that the AIP was an independent predictor for early-onset PSD. AIP was utilized as a tertile-based categorical variable; after adjusting for confounding variables, elevated AIP levels remained an independent risk factor for early-onset PSD. Additionally, in the subgroup analysis of this study, all interaction terms did not show significance (P>0.05), indicating that the effect of AIP on early-onset PSD risk was consistent across different subgroups, indicating that our study conclusion is robust and applicable to a wide range of populations. Previous studies have shown that gender, age, lesion location, and stroke severity are associated with post-stroke depression (4). However, there was no significant relationship between gender, age, or lesion location and early-onset PSD in our study. We believe that the variances in the ethnicity of the research populations, the small sample sizes, the medication status, and the severity of the condition may be the causes of the discrepancies between various studies. Finally, the AIP demonstrated commendable early-onset PSD discriminative capability with AUC values of 0.785. These data may suggest that the AIP serves as a biomarker for early-onset PSD. Our findings enhance the comprehension of AIP's role in early-onset PSD and offer new insights into therapeutic strategies.

The mechanism remains unknown for AIP increasing the risk of early-onset PSD. We believe that this phenomenon can be explained by a number of possible mechanisms. Firstly, the size of small, dense LDL particles, a subcomponent of LDL with pro-inflammatory and pro-atherogenic characteristics, is mostly represented by AIP (41). Higher AIP may affect the prognosis of ischemic stroke by promoting plaque inflammation and susceptible plaque rupture (20). Secondly, a crucial lipid component of biological membranes, cholesterol is necessary for both preserving the fluidity of cell membranes and guaranteeing appropriate synaptic activity (42, 43). High lipid consumption leads to the buildup of glial cells, which negatively impacts hippocampal neurons and heightens the likelihood of depression (44). Reduced metabolism of serum cholesterol may directly affect brain lipid levels and cell membrane fluidity, which in turn may disrupt serotonergic neurotransmission (45, 46). The pathophysiologic route that depression and PSD share may be abnormal lipid metabolism. The finding that both decreased HDL-C levels and increased LDL/HDL ratios may serve as risk factors for PSD provides evidence in favor of the link between abnormal lipid metabolism and PSD (31). Thirdly, a recent study also found that a higher AIP index was linked to early neurological deterioration (END) in AIS patients who had mechanical thrombectomy treatment for an urgent large vessel occlusion (47). END was defined as an increase of ≥ 4 points in NIHSS within 24 hours. Moreover, earlier studies have found strong correlations between AIP and several associated conditions, such as coronary heart disease, diabetes mellitus, and hypertension, all of which have been identified as significant risk factors for END (48). Depression may increase the risk of cardiovascular disease by accelerating the development of atherosclerosis through endothelial dysfunction or escalating inflammation (49). The substantial detrimental effects of depression on the incidence and course of cardiovascular illnesses have also been demonstrated by numerous research studies (23, 50). Treating depression may improve the prognosis for coronary heart disease (51, 52). According to earlier research, AIP is linked to the prevalence of depression, and higher AIP levels raise the risk of developing depression (21). AIP can also be employed as a predictor of the severity of coronary artery disease and as a possible biomarker for the early detection of cardiovascular disease events (53, 54). In addition, elevated AIP and atherosclerotic indices, which raise atherosclerotic risk, are partially responsible for the co-occurrence of mood disorders and cardiovascular disease (55). Consequently, atherosclerosis induced by elevated AIP serves as an intermediary in the development of mood disorders, as well as cardiovascular and cerebrovascular diseases.

This study has the following limitations: (1) This study was a single-center study with a relatively small sample size and involved only Chinese patients, indicating the presence of potential inherent biases; therefore, the study findings still need to be further confirmed by multi-center and large-sample clinical studies; (2) the study excluded patients who experienced severe aphasia, coma, or dementia while in the hospital, which could have influenced the prevalence of early-onset PSD; (3) several risk factors that could influence depressive episodes, including mounting life stress, educational background, and social support were not included in our study; (4) conclusions from short-term observational studies may not be comprehensive enough; and (5) there were no blood test data during follow-up and dynamically observing changes in AIP would be more valuable for predicting early-onset PSD.

## Conclusion

Our study indicated that AIP may be an independent risk factor for early-onset PSD and can be used as a predictive indicator. Finding a link between AIP and early-onset PSD has significant therapeutic implications for early-onset PSD screening, etiology study, treatment plan development, and prognosis assessment. It is anticipated that integrating AIP into evaluation and treatment plans will enhance patients' overall clinical results. However, further studies are needed to clarify the exact mechanism of AIP in the pathogenesis of early-onset PSD.

## Data Availability

The raw data supporting the conclusions of this article will be made available by the authors, without undue reservation.

## Supplementary material

Supplementary Figure 1 Subgroup analyses of AIP and early-onset PSD.
