# Supplementary figures and images for: Association of atherogenic index of plasma with early-onset post-stroke depression: a prospective study
Source: Front Psychiatry. 2025 Jul 25;16:1563289. doi: 10.3389/fpsyt.2025.1563289 (PMC12331589; doi:10.3389/fpsyt.2025.1563289)

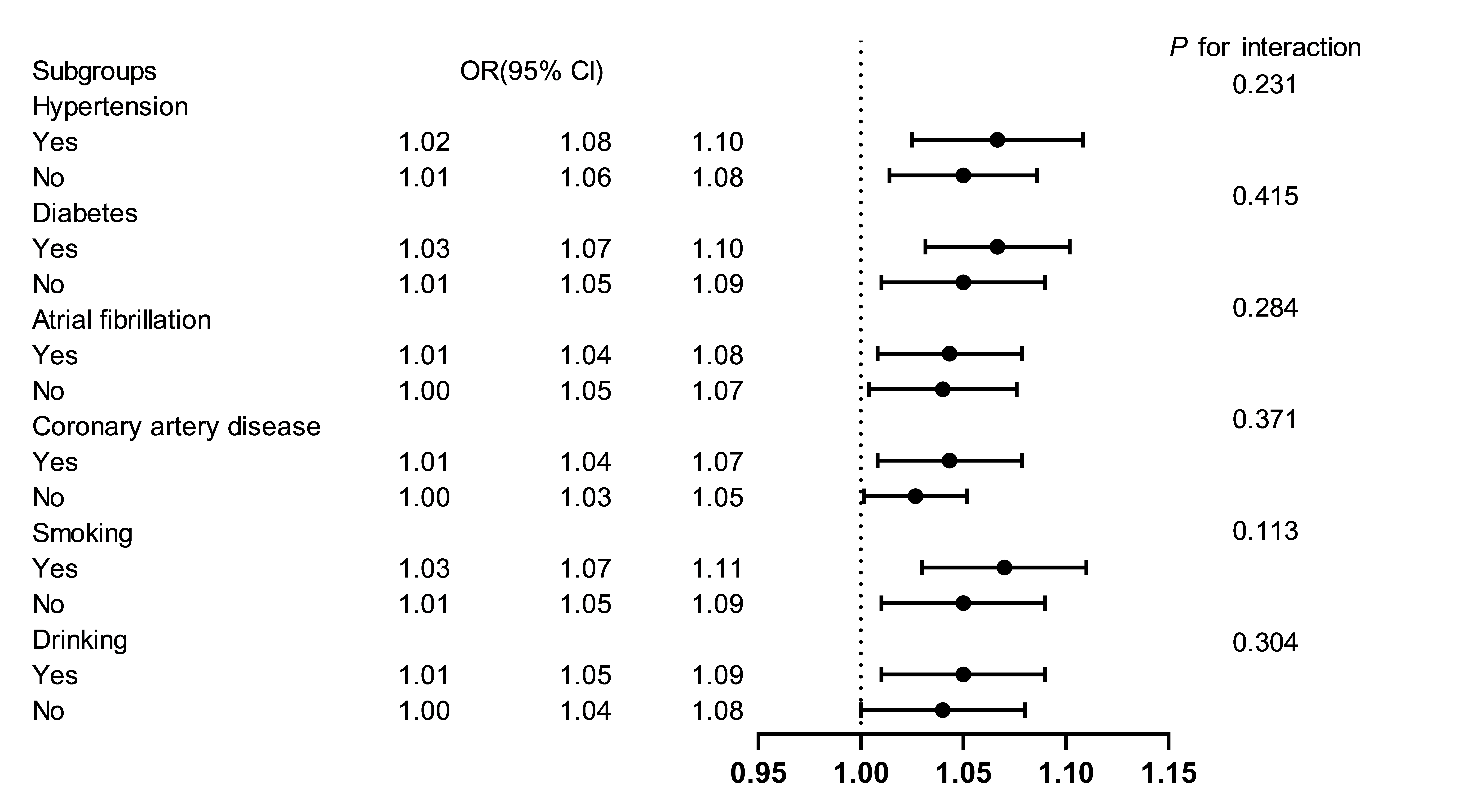

Supplement: Supplementary Figure 1 — Subgroup analyses of AIP and early-onset PSD. [file Image1.tif]
